# Impact of Sports Medicine and Orthopedic Surgery Rotations on Musculoskeletal Knowledge in Residency: An Update and Longitudinal Study

**DOI:** 10.7759/cureus.32830

**Published:** 2022-12-22

**Authors:** William Denq, Allison D Lane, Alex Tomesch, Sara Zagroba, Thomas M Cahir, Anna Waterbrook

**Affiliations:** 1 Emergency Medicine/Sports Medicine, University of Arizona College of Medicine - Tucson, Tucson, USA; 2 Emergency Medicine/Sports Medicine, University of Missouri, Columbia, USA; 3 Health Sciences, University of Arizona College of Medicine - Tucson, Tucson, USA

**Keywords:** assessment, freedman, sports medicine, orthopedics, emergency medicine, curriculum, education, musculoskeletal knowledge

## Abstract

Introduction

Musculoskeletal (MSK) complaints and injuries account for a significant percentage of presenting chief complaints to the emergency department in the United States (US). Despite the prevalence of disease and economic impact on the US healthcare system, there is a documented deficiency in MSK education at all training and practicing levels in the US medical system. The purpose of this study is to determine MSK knowledge acquisition after an orthopedic or primary care sports medicine (PCSM) rotation in three emergency medicine (EM) residency programs at a single institution.

Methods

A total of 115 EM post-graduate year 1 (PGY-1) residents participated in and completed this study over five academic years. Based on existing residency program curricula, the participants were categorized into two groups. One group completed a traditional four-week Orthopedic Surgery rotation and the other group completed a four-week Sports Medicine rotation. The validated written Freedman and Bernstein MSK examination (FB-MSK) was administered to all participants at the start of residency and at completion of their rotation. Fifty-nine of the participants participated in a longitudinal secondary study over five academic years. The FB-MSK was offered to all participants every year following the completion of their rotation during their residency.

Results

Post-rotation scores improved regardless of which group the resident belonged to. The orthopedic group improved an average of 3.11 points (p = <0.0001, CI 2.39 to 3.82) and the average improvement in the PCSM group was 3.97 points (p = <0.0001, CI 2.81 to 5.83). The post-rotation scores were similar regardless of the group (p = 0.4287, CI -0.73 to 1.70). The amount of improvement in scores between the two groups was not statistically significant (p = 0.209, CI -0.49 to 2.21). Of the longitudinal participants, PGY-3+ significantly scored higher than PGY-1 (p = 0.0325, 95% CI 0.165 to 3.658).

Conclusion

Regardless of rotation type, MSK knowledge acquisition appears to significantly improve. EM senior residents demonstrate significant MSK knowledge acquisition during residency. Further studies on a multi-institutional level are needed to account for MSK curriculum variability in residency programs.

## Introduction

Musculoskeletal (MSK) complaints and injuries are a common reason patients seek medical care and make up 14.3 to 18.7% of presenting chief complaints to the emergency department (ED) in the United States (US) [1,2]. These problems can lead not only to significant morbidity amongst patients, but also have a large economic impact on the US healthcare system [3]. Despite the prevalence and high economic burden of these disorders, previous studies describe a deficiency in MSK education at all training levels and among practicing physicians in the US medical system [4-11]. Emergency physicians (EPs) are no exception. Previous studies reported only 61% of EPs passed a validated MSK examination [5] and a recent study demonstrated 56.1% of new Emergency Medicine (EM) residency graduates felt “not at all prepared” or “somewhat prepared” for MSK complaints [12]. Despite this well-documented deficit, there have been few interventions to improve MSK education for EM trainees and practicing EPs. In fact, the Accreditation Council for Graduate Medical Education (ACGME) does not require nor mention MSK curricula for EM Residency Program accreditation [13]. The only criterion is performing a minimum of 10 dislocation reductions. The American Board of Emergency Medicine (ABEM) provides a recommended, but not mandated, core model of EM clinical practice [14]. This model is intended to guide residency curricular development, but only mentions MSK topics within the confines of traumatic disorders.

There have been a few attempts to address this educational deficiency among EPs. The American Society for Sports Medicine (AMSSM) recently published recommended curricular guidelines for MSK and Sports Medicine in EM residency [15]. A study by Waterbrook et al. reported the incorporation of a primary care sports medicine (PCSM) rotation for EM residents. They found the rotation yielded higher resident satisfaction compared to a traditional orthopedic rotation [16]. We previously reported on the level of EM resident MSK knowledge acquisition before and after a PCSM rotation or traditional orthopedic rotation using a validated MSK examination by Freedman and Bernstein (FB-MSK) [17]. The study showed significant MSK knowledge acquisition in both groups but no difference in the level of knowledge acquisition after completion of either traditional orthopedic surgery or PCSM residency rotation. The purpose of this study is to update our initial report with three additional years of data, provide a longitudinal perspective, and expand the current body of literature regarding MSK knowledge acquisition before and after a curricular intervention.

## Materials and methods

We recruited post-graduate year 1 (PGY-1) residents from two categorical Emergency Medicine residency programs and one combined Emergency Medicine/Pediatric program over five academic years (2015-2020). The study protocol divided participants into two groups based on their designated residency program and corresponding curriculum. One group completed a traditional four-week Orthopedic Surgery rotation and the other group completed a four-week PCSM rotation. To note, we previously reported years 2015 to 2017 and included the dataset to power this study [17].

The Orthopedic Surgery rotation occurred at a level-one trauma center with the Department of Orthopedic Surgery’s inpatient and consult service. Study participants assigned to the traditional orthopedic rotation were responsible for inpatient and emergency department consults including morning rounds, management of post-operative orthopedic patients, acute fracture management, and emergency department procedures such as reductions and arthrocentesis.

The PCSM rotation occurred at an outpatient Sports Medicine clinic and level-four trauma center. The curriculum included attending clinic with Primary Care and Orthopedic Sports Medicine physicians, performing procedures such as arthrocentesis, didactic experiences, sideline and event coverage, and an on-call schedule to assist with reductions in the Emergency Department.

The FB-MSK was precepted and administered to all participants at the start of residency and then immediately after completion of their designated rotations during PGY-1. The test was also administered annually, at year-end, to all residents. The FB-MSK has 25 short-answer questions with a passing score defined as 73.1% or above, based on the original Freedman-Bernstein study [17].

The pre- and post-test scores for all participants were entered into an Excel spreadsheet and statistical analysis was performed using the R program [18]. A paired t-test and chi-square test were performed to determine if there were significant differences of the pre- and post-test scores and pass rates between the two rotations. End-of-year test scores were also analyzed for longitudinal differences throughout residency years.

This project was reviewed by the University of Arizona Institutional Review Board and approved as an exemption, IRB 00000261.

## Results

A total of 124 EM PGY-1 residents were initially recruited to participate in the study. Nine were unable to complete the post-test and were not included in the analysis. Four residents were unable to complete an orthopedic rotation due to the COVID-19 pandemic, four were lost to attrition, and one was unable to complete a post test. All 115 residents included in the study completed the FB-MSK at the onset of their first year and completed it again at the end of their PCSM or orthopedic rotation. Demographic data of the residents are in Table 1.

**Table 1:** Demographic data of the study participants included in analysis PCSM: Primary Care Sports Medicine; MSK: Musculoskeletal; FB-MSK: Freedman and Bernstein Musculoskeletal Examination

	Orthopedic rotation N=84 (%)	PCSM rotation N=31 (%)
Medical degree	84 (100)	31 (100)
Allopathic	71 (85)	29 (93.5)
Osteopathic	13 (15)	2 (6.5)
MSK rotation in medical school	37 (44)	13 (42)
Considering sports medicine as a subspecialty	15 (18)	5 (16)
Previous MSK certification	0 (0)	0 (0)
Passed FB-MSK pre-rotation	18 (22)	8 (26)
Passed FB-MSK post-rotation	47 (56)	22 (71)

The mean pre-rotation scores of the two groups did not differ statistically. The orthopedic group had an average score of 15.56 and the PCSM group had an average score of 15.19 (p = 0.5714, CI -1.68 to 0.93). The mean post-rotation scores of the two groups did not differ statistically either. The orthopedic group had an average score of 18.67 and the PCSM group had an average score of 19.16 (p = 0.4287, CI -0.73 to 1.70). The improvement in both groups was statistically significant with the average improvement in the orthopedic group being 3.11 points (p = <0.0001, CI 2.39 to 3.82) and the average improvement in the PCSM group being 3.97 (p = <0.0001, CI 2.81 to 5.83). The amount of improvement in scores between the two groups was not statistically significant (p = 0.209, CI -0.49 to 2.21). Although 71% of the PCSM group passed the post-test while 55% of the orthopedic group passed, this was not statistically significant (p = 0.4421). The initial average score of the PCSM group was lower than the orthopedic group (15.19 vs 15.56). The average post-test score of the PCSM group was not significantly higher than the orthopedic group (19.16 vs 18.67; p = 0.658, CI -1.98 to 1.26).

A subset of study participants (n = 54) was followed longitudinally. Follow-up was achieved in 35 residents in the orthopedic group and 19 residents in the PCSM group. Seventy residents were lost to follow-up for longitudinal data out of the initial 124. PGY-3+ residents had statistically significantly improved scores compared to PGY-1 residents (17.87 vs 15.95, p = 0.0325, 95% CI 0.165 to 3.658). None of the other years outperformed the PGY-1 group at a statistically significant level. PGY-1 vs PGY-2 (15.95 vs 17.46, p = 0.0981, 95% CI -0.29 to 3.31) and PGY-1 vs PGY-3 alone (15.95 vs 17.43, p = 0.1132, 95% CI -0.367 to 3.27) were not statistically significant. Additional data is located in Appendix A.

## Discussion

Training and acquisition of MSK knowledge is reported to be insufficient during the career of an EP. To our knowledge, this is the first report to demonstrate there is a significant improvement in MSK knowledge acquisition by the end of residency compared to the start of training. Whether the etiology for this acquisition is due to knowledge retention, outside curricular intervention, clinical exposure, or otherwise is unclear.

Post-rotation scores significantly improved after curricular intervention, regardless of rotation type. This result continues to suggest the rotations’ effectiveness at MSK knowledge acquisition. The PCSM rotation represents a viable alternative to the traditional orthopedic rotation. We previously reported that the PCSM rotation resulted in higher resident satisfaction compared to the traditional orthopedic rotation [16]. Although it is unclear if continuation of this study would result in statistically significant differences between the two rotations, further investigation can delve into different knowledge acquired from the rotations. This may aid in the development of a more standardized MSK curriculum among EM residency programs.

The MSK and SM model curricular guidelines for EM residents released by AMSSM provides a foundation for which to build a standardized MSK curriculum. Despite varied institutional cultures and resource availability at programs across the nation, this study demonstrates that exposure to an MSK rotation may help improve the knowledge deficit. In programs where the orthopedic surgery department has a strong presence, collaboration or a longitudinal focus on developing MSK knowledge may improve the comfort and knowledge base of residency graduates.

A multi-specialty approach to developing MSK curriculum has been reported by several studies. Battistone et al. reported a significant improvement in resident ability to evaluate and manage MSK complaints after the institution of a multi-specialty curriculum [19]. Gil et al. reported significant improvement in residents in nine core content areas after the institution of an orthopedic rotation developed by EM and orthopedics [20].

Support from the ACGME to require more than a procedural skill in fracture reduction may help with MSK knowledge acquisition. Requiring a fundamental understanding of traumatic and atraumatic MSK pathology should be a part of EM curricular guidelines. A categorical list of MSK pathologies and diagnostic and treatment methodologies should be developed and maintained by ACGME and ABEM. Coordinated support should be provided from recognized societies in Emergency Medicine, Orthopedics, and Sports Medicine. Longitudinal courses, blocks, small group learning, online modules, asynchronous learning, near-peer learning based on AMSSM curricular guidelines are several potential solutions. This, in turn, may increase EPs comfort in managing MSK complaints and improving patient outcomes. Further studies are needed to test this hypothesis.

Several challenges and limitations exist in this study. Although the study measured data across three training programs, they all originate from a single institution, which may limit the result’s generalizability. Without data from other institutions, it is possible that any exposure to experts with MSK experience and knowledge will increase MSK knowledge acquisition. Without multiple versions of the FB-MSK or question randomization, the post-test results may be skewed from pre-test knowledge retention. Despite the national validation of the FB-MSK, it is not specifically validated for EPs and was validated in 1998. The exam assesses MSK knowledge, but not physical examination or procedural skill. It also does not follow recommended guidelines released by ABEM or AMSSM. Recently, Cummings et al. developed the MSK-30 and found evidence of validation in primary care medicine residents and graduating medical students [21]. However, at the time of this report, there are no known validated exams published for emergency medicine physicians.

## Conclusions

This study statistically bolsters the hypothesis that an MSK curricular intervention regardless of rotation type helps improve the knowledge base of EM residents. EM residents acquire significant MSK knowledge during residency. Further research to assess MSK knowledge in EM residents, report on MSK knowledge acquisition, or development of innovative MSK curricular interventions at other institutional EM programs is needed.

## Appendices

Appendix A. An aggregation of all data from the longitudinal study is summarized in Table 2 and Table 3.

**Table 2:** Longitudinal test scores based on resident year PGY: Post-Graduate Year

	PGY-1	PGY-2	PGY-3	PGY-3+
Number of participants	16	31	25	30
Average score	15.95	17.46	17.43	17.87
Standard deviation	2.86	2.93	2.85	2.77

**Table 3:** Longitudinal test scores compared by year of residency PGY: Post-Graduate Year; CI: Confidence Interval

	Average Scores	P-value	95% CI
PGY-1 vs PGY-2	15.95 vs 17.46	0.0981	-0.29 to 3.31
PGY-1 vs PGY-3	15.95 vs 17.43	0.1132	-0.367 to 3.27
PGY-1 vs PGY-3+	15.95 vs 17.87	0.0325	0.167 to 3.658
PGY-1 vs All	15.95 vs 17.52	0.0527	-0.019 to 3.153
